# How do attitudes shape protective practices against the Asian tiger mosquito in community gardens in a nonendemic country?

**DOI:** 10.1186/s13071-022-05520-3

**Published:** 2022-11-22

**Authors:** Pénélope Duval, Claire Valiente Moro, Christina Aschan-Leygonie

**Affiliations:** 1 UMR 5557 Ecologie Microbienne, Univ Lyon, Université Claude Bernard Lyon 1, CNRS, INRAE, VetAgro Sup, Bâtiment Dubois 43 Boulevard du 11 Novembre 1918, 69622 Villeurbanne, France; 2 UMR 5600 CNRS Environnement Ville Société, University of Lyon, Université Lumière Lyon 2, 69007 Lyon, France

**Keywords:** Asian tiger mosquito, KAP survey, Protective practices, Nonendemic country, Green urban area

## Abstract

**Background:**

The Asian tiger mosquito *Aedes albopictus* is responsible for the transmission of many arboviruses worldwide and is well adapted to thrive in urban environments. In mainland France, a nonendemic area, this mosquito is responsible for several autochthonous and imported cases of chikungunya and dengue each year. Better management and prevention of mosquito-borne disease transmission in nonendemic areas is thus of global concern. In this context, the aim of this study was to provide a better understanding of mosquito–human interactions as well as human behavior and beliefs in regard to this mosquito species in urban areas.

**Methods:**

We focused on people who participate in community gardens, which are increasingly popular initiatives in metropolitan France and are conducive to the development of tiger mosquitoes. To evaluate community gardeners’ knowledge and practices in relation to mosquito management and control, we conducted a knowledge, attitude, and practice (KAP) survey.

**Results:**

In contrast to previous KAP studies, we showed that attitudes, more than knowledge, influence the practices of community gardeners in relation to mosquitoes. Interestingly, all gardeners who participated in the survey were concerned about the Asian tiger mosquito and were motivated to incorporate mosquito control methods in their gardens. Moreover, mosquitoes were perceived as nuisances rather than disease vector species. A change in community gardeners’ perceptions could facilitate more appropriate behavior to control this species.

**Conclusions:**

This survey reveals the lack of knowledge and awareness of good practices for the efficient control of the Asian tiger mosquito in green urban areas.

**Graphical Abstract:**

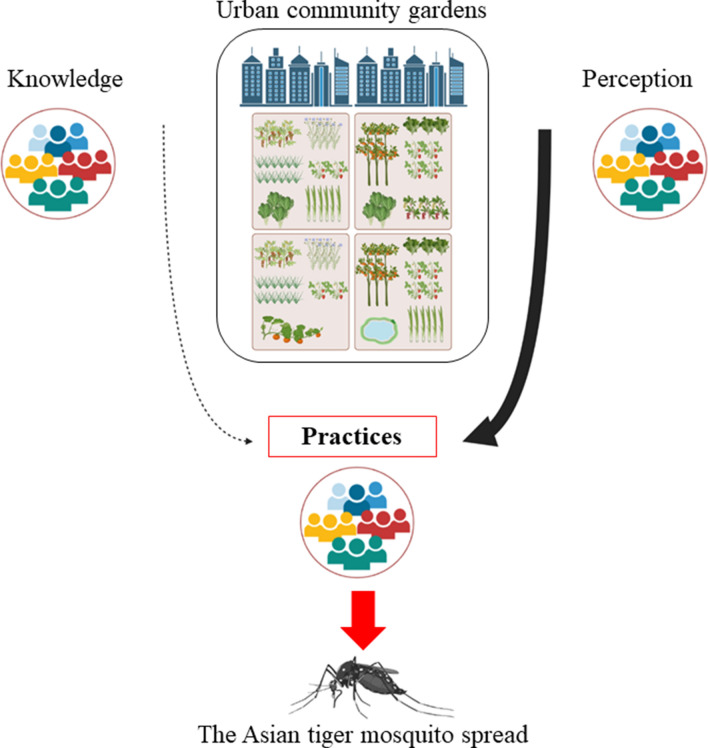

**Supplementary Information:**

The online version contains supplementary material available at 10.1186/s13071-022-05520-3.

## Background

The Asian tiger mosquito *Aedes albopictus* is one of the world's most invasive species [1]. This mosquito species, which originates in temperate areas in southeastern Asia, has expanded on all continents except for Antarctica since the second half of the twentieth century [2]. Since its introduction in Europe via the transportation of goods and increased international travel, the presence of this mosquito species has been recorded in an increasing number of European countries, including mainland France, where it was detected for the first time in 2004. Human transportation and land use have been shown to be key factors for *Ae. albopictus* dispersal and the establishment of new mosquito propagules across the territory [3]. Due to its expansion in the French territory, a parliamentary report stated in 2020 that the presence of the Asian tiger mosquito constitutes a major health risk in the decades to come [4]. This mosquito species is a competent vector for many arboviruses, such as dengue (DEN), chikungunya (CHIK), and Zika (ZIK) viruses, as well as filarial nematodes [5]. Since 2010, it has been responsible for a growing number of autochthonous cases of CHIK and DEN in mainland France (Calba et al. [6]; Durand et al. [7]) as well as imported cases. For instance, Santé Publique France reported 109 DEN, 12 CHIK, and one ZIK confirmed imported cases from May 1 to June 7, 2019, in mainland France [8]. Beyond these health issues, this mosquito species is an aggressive daytime biter with a social impact because it can significantly alter human behavior and activity [9].

Previous studies have shown that the development and survivorship of *Ae. albopictus* are reinforced in cities compared to rural areas [10, 11]. Urban environments are particularly conducive to mosquito development and survival for several reasons. First, they provide numerous artificial water containers (gutters, tires, flat roofs, flowerpots, cemetery urns, etc.) and storm water management structures (rainwater network, gully or combined sewer) that can be used as larval habitats as *Aedes* mosquito larvae require standing water to complete their life-cycle. Second, densely populated urban areas provide good opportunities for blood feeding [12]. Finally, urban densification generates urban heat islands (UHIs) that are characterized by higher temperatures compared to surrounding areas [13]. The increase in surface temperature in urban areas has a direct impact on mosquitoes as it enhances their chances of survival by stimulating foraging and egg-laying behavior as well as shortening larval development and the duration of the extrinsic incubation period of the pathogen [14–17]. All these factors (human population density, UHIs, larger numbers of artificial water containers) increase the risk of arbovirus exposure in urban environments, a problem due to growth as a result of the urban densification policies that are currently implemented in France.

Numerous studies show that a significant increase in green areas and vegetation in cities is an efficient strategy to combat UHIs and reduce cities’ vulnerability to heat and the effects of climate change [18–20]. Consequently, current policies favor nature in the city by developing green spaces as well as roof and wall gardens and, more generally, by avoiding concretization. In recent years, community gardens (e.g., family gardens, shared gardens, and integration gardens) have spread in cities all over Europe [21]. Various stakeholders contribute to the establishment of community gardens, including municipalities, nonprofit organizations, and private companies, and they are often linked with citizens’ initiatives. These gardens have multiple beneficial impacts on health and well-being, such as (i) social impact (e.g., locations for conviviality, tools for reintegration), (ii) political impact (e.g., collective production and discussion), (iii) environmental impact (e.g., awareness of environmental problems, biodiversity, and resources), (iv) economic impact (e.g., food production and exchange), and (v) health impact (e.g., increasing physical activity) [22, 23]. Community gardens are increasingly popular initiatives in France. In the Ile de France region, the number of collective gardens increased from fewer than five in 2000 to approximately 100 in 2021 [24]. Although no database referencing collective gardens in mainland France is available to date, it is assumed that their number will increase significantly in the coming years, especially in the largest cities. An example is the city of Lyon, which has been promoting the development of community gardens for the past 20 years and encourages its residents to green the city. Currently, there is a multiplication of participatory gardening programs [25]. The growing demand for urban gardens is partly explained by the recent COVID-19 pandemic. Lockdowns and social restrictions have changed people’s behaviors and attitudes related to urban green spaces [26]. In addition, an increasing interest in urban vegetable gardens and food self-sufficiency has been observed [27]. However, it should be noted that along with the benefits listed above, community gardens increase the number of aquatic containers (watering cans, flower buddies, or rainwater collectors), which are conducive to tiger mosquito development [28].

Due to close links between *Ae. albopictus* and humans, human practices and human knowledge may significantly influence the mosquito life-cycle in urban green areas. Here, we focus on French community gardens in metropolitan Lyon for three main reasons: (i) the *Ae. albopictus* mosquito species has progressively been implemented in this area since 2012, (ii) imported cases of arbovirus infections are reported every year in the Rhône region, with the first autochthonous case of DEN reported in 2019 [29], and (iii) for several years, the Lyon metropolis has encouraged the establishment of collective gardens throughout its territory [30]. By conducting a quantitative survey, we will gain a better understanding of community gardeners’ knowledge, attitudes, and practices (KAP) in relation to mosquito management and control for the first time in mainland France to develop an adaptive awareness plan. This survey also allowed us to measure the level of motivation of gardeners regarding their active participation in the fight against the Asian tiger mosquito. The outcomes of the investigation are particularly important as we are currently conducting a study based on the use of biological methods for the control of this mosquito species in community gardens within the Lyon metropolis. The current survey is part of this larger study.

## Methods

### Study area

**Fig. 1:**
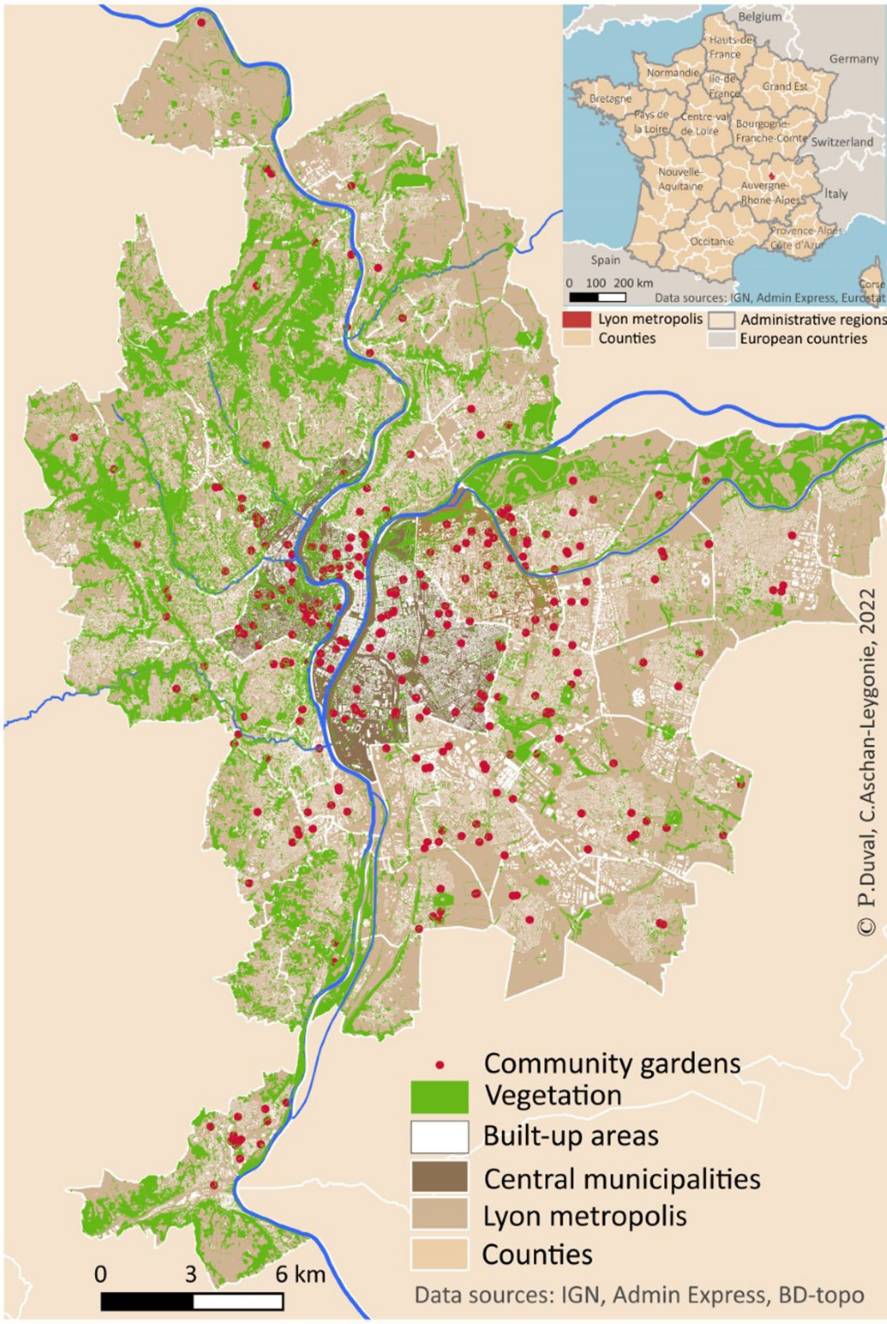
Distribution of the community gardens in the Lyon metropolis (**a**) and location of Lyon in France (**b**)

**Fig. 2:**
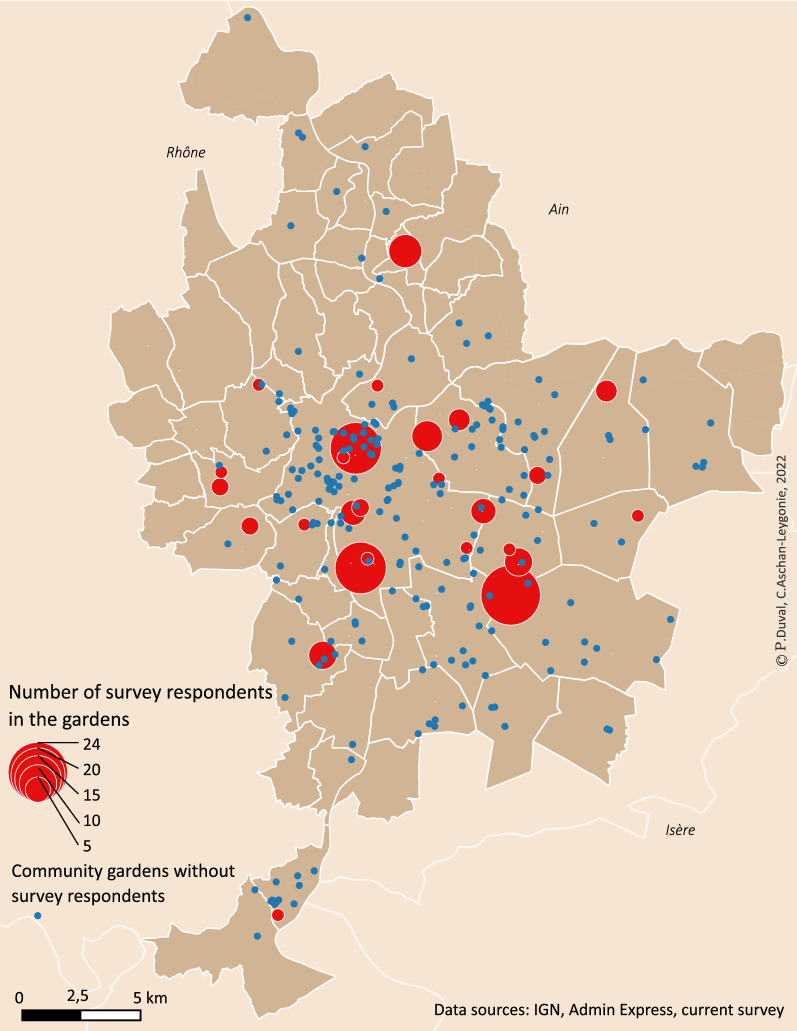
Number of survey respondents in the community gardens

The survey was conducted among community gardeners in the Lyon metropolis, which is the second largest French city located in the region of Auvergne-Rhône-Alpes in southeastern France (Fig. 1). The Lyon metropolis had 1,411,571 inhabitants in 2019 spread over 59 municipalities in an area of 5337 square kilometers. The population density in the metropolitan area is 26,449 inhabitants per square kilometer, but there is high variability between the 59 municipalities. The urban core, with a population density of approximately 10,500 inhabitants per square kilometer, is roughly situated within the two central municipalities (Lyon and Villeurbanne). In the outer periphery, the built environment is less dense, and the share of green areas is increasing (Fig. 1). On the whole, the Lyon metropolis accounts for 40% of green areas. At the beginning of this study, there was no exhaustive inventory of community gardens in the metropolis. The local association of community gardens (*le Passe Jardin*) held the most complete inventory; in 2020, it had 200 registered gardens in its database. This inventory distinguishes between shared gardens, family gardens, and integration gardens but does not account for the number of regular gardeners in each garden. As a first step of the survey, we completed this database by various methods: information requests from municipalities, analysis of satellite images, and internet searches. This painstaking research resulted in a geographical database (using QGIS [31]) with 288 collective gardens located within the metropolitan area of Lyon (Fig. 2). The surveyed respondents were spread over 83 different community gardens located in 25 different municipalities of the Lyon metropolis. The target population in the survey was gardeners in the 288 community gardens that we identified in 2020. Following observations in the field and questioning of gardeners, we made a rough estimate that suggested that there were between 10 and 30 regular gardeners in most gardens.

### KAP survey

We implemented a KAP survey to provide representative data on the knowledge, attitudes, and practices of community gardeners in relation to the Asian tiger mosquito. The quantitative survey included 77 questions divided into closed-ended and open-ended questions. For closed-ended questions, survey participants had the option to select several answers. The complete questionnaire is presented in the (Additional file 1: Table S1). The first topic, “frequentation and practices in collective gardens,” consisted of 34 questions and aimed to identify the garden and the respondent’s role in the specific community garden as well as the gardener’s activities and practices regarding cultivation in the garden (for example, pesticide use, watering, period of presence in the garden). We specifically asked about the layout of the parcels and the watering practices as the type of irrigation directly affects the presence and number of potential mosquito breeding sites in the gardens. In the second topic, 19 questions were related to general “nuisances” and aimed to identify different types of nuisances in gardens. At this stage of the survey, there was no mention of the Asian tiger mosquito to avoid influencing the respondents. In the third topic, namely, “knowledge of the Asian tiger mosquito,” the respondents were given 11 questions related to the mosquito’s physical characteristics, life-cycle, and place in the ecosystem. In the fourth topic, seven questions were related to “biological control methods” and concerned means of mosquito control in community gardens and knowledge of biological control methods. This section also addressed the issue of the respondent’s personal motivation to become an active partner in the implementation of such control methods in the collective garden. The last topic, which included eight questions on “sociodemographic data,” aimed to describe the respondents’ profiles, such as gender, age, employment status, profession, and education. A pilot version of the questionnaire was sent to 10 community gardeners to check its validity (framing of the questions, overall structure, and sequences, etc.) and to ensure that the questionnaire length was compatible with the estimated duration it would take a respondent to complete the questionnaire. The final version was sent by email to all community garden contacts (*n* = 164), to the local association *le passe jardin*, and to all municipalities of the Lyon metropolis (*n* = 59), which agreed to dispatch the questionnaire to the members of the community gardens. We chose an internet-based survey, which is an efficient means to reach a large pool of potential participants who are both geographically dispersed within the metropolitan area and otherwise difficult to access (due to high variability of daily, weekly, and seasonal presence of gardeners in the community gardens). In addition, this type of survey is particularly efficient in regard to data collection and processing. With the advent of the COVID-19 pandemic, this choice proved to be particularly appropriate as the distribution of the questionnaire by mail was performed between March and July 2021. We obtained a total of 265 questionnaires, of which 255 were completed on the internet by the respondents, and 10 were collected directly in the field in one garden.

### Ethics statement

The survey was developed using the software Sphinx IQ 2 at the University of Lyon secured server. The anonymity of each participant was respected. Each participant was associated with a code for data collection and analysis.

### Data analysis

Statistical descriptive analyses were used to identify the respondents’ characteristics, their frequencies and practices in gardens, and their perception of nuisance, biological control, and tiger mosquitoes. Responses are represented as quantities and percentages. We also conducted bivariate analyses based on the chi-square test of independence to determine whether there was a statistically significant relationship between categorical variables. This nonparametric test provides information not only on the significance of the general relationship between two variables but also on which categories account for any differences found between the theoretical and the observed situations. The aim using the chi-square test was, on the one hand, to identify statistical relations within a 95% confidence interval and, on the other hand, to establish which categories were significantly different from the expected values. Explanatory variables included sociodemographic parameters, frequentation and practices in collective gardens, nuisances, and the Asian tiger mosquito.

## Results

### Sociodemographic characteristics and motivations of the respondents

The sociodemographic characteristics of the surveyed gardeners are shown in Table 1. Overall, 51.4% of the 265 community gardeners who participated in this survey were female, and 48.6% were male. Most participants were in the range of 50–65 years old, and most of the gardeners were either professionally active (51.4%) or retired (39.6%). A large majority of the respondents (64.9%) had a high education level, above a high school diploma. Most respondents joined the garden between 1 and 5 years ago (46%), and in general, they spent more than 2 h (53.1%) each time in the garden (Table 2). A total of 19% of the respondents were activity leaders and/or initiators of the community garden. Gardeners were mainly motivated to join a community garden for the pleasure of gardening (79.6%), to grow fruits and vegetables themselves (71.36%), or to create social links (62.8%).

**Table 1:** Sociodemographic characteristics of the survey respondents

Category	No. of respondents	Proportion within each category (%)
Gender		
Female	114	51.4
Male	108	48.6
Age group		
18–35	15	6.8
35–50	65	29.3
50–65	75	33.8
> 65	53	30.2
Profession		
Active	115	51.4
Retired	88	39.6
Looking for a job/not working	9	4.1
In professional training	4	1.9
Other	6	2.7
Education level		
Below high school diploma	32	14.4
High school diploma	43	19.4
Above high school diploma	144	64.9
Other	3	1.4

**Table 2:** Responses of gardeners regarding attitudes and practices in the community garden

Category	No. of respondents	Proportion within each category (%)
When did you join a community garden for the first time?		
< 1 year	29	10.9
Between 1 and 5 years	122	46
Between 6 and 10 years	53	20
> 10 years	61	23
How long do you stay on average in community garden?		
Between 30 min and 1 h	33	13.5
Between 1 and 2 h	78	31.8
> 2 h	130	53.1
What motivates you to participate in a community garden? (multiple answers allowed)		
For the pleasure of gardening	199	79.6
Growing your own fruits and vegetables	179	71.6
Enhancing social connections	157	62.8
Participating in outdoor activities	123	49.2
Participating in physical activities	78	31.2
Showing children how to garden	73	29.2
In this garden, what activities do you practice? (multiple answers allowed)		
Growing fruits and/or vegetables	235	91.1
Planting ornamental plants and/or flowers	176	68.2
Participating in workshops	109	42.2
Performing do-it-yourself activities	36	14

### Knowledge, attitudes, and practices in community gardens

*Knowledge of the Asian tiger mosquito*. To assess community gardeners’ knowledge of mosquitoes and their proliferation control, the participants answered a number of questions about mosquito characteristics (e.g., life-cycle, physical aspect, what mosquitoes feed on) and biological methods that can be used against mosquitoes. Table 3 summarizes mosquito knowledge among the respondents. All respondents had heard about the Asian tiger mosquito, and 85.7% thought that they would be able to identify this unique species among other mosquito species. However, only 32.8% of the respondents knew that the Asian tiger mosquito has a dorsal line that is the distinctive characteristic of the species. In addition, 94.7% of respondents knew that the Asian tiger mosquito breeds in medium and small stagnant water reservoirs, but 81.6% also incorrectly believed that this mosquito breeds in other inappropriate areas, such as vegetation. Only 20.5% of the respondents had ever heard of biological control methods. Among these respondents, 31.3% were unable to explain the significance of biological control, and very few respondents (*n* = 9) clearly had knowledge of this type of pest control and could explain different methods, such as mosquito predator reintroduction or the use of *Bacillus thuringensis toxin*, i.e., a toxin commonly used against mosquitoes. A total of six gardeners were confused about mechanical methods, such as the elimination of standing water containers.

**Table 3:** Mosquito knowledge in the community gardens (multiple answers were allowed for these questions)

Category	No. of respondents	Proportion within each category (%)
Garden pests		
Mosquitoes	172	72.3
Aphids	168	70.6
Slugs	157	66
Snails	123	51.7
Colorado potato beetles (doryphore)	108	45.4
Birds	101	42.4
Rodents	83	34.9
Ticks	7	2.9
The Asian tiger mosquito is		
A mosquito species that infects humans and animals	201	88.5
A pest (that bites)	161	70.9
An invasive species	131	57.7
Very abundant in the French metropolitan area	120	52.9
A useful species in the food web	16	7
What is a characteristic of the Asian tiger mosquito?		
The stripes on the legs	142	76.3
The size	100	53.8
The dorsal line	61	32.8
Diurnal activities	39	21
The Asian tiger mosquito develops in		
Medium and small stagnant water reservoirs	215	94.7
Large water bodies (lakes, for example)	90	39.6
Vegetation	64	28.2
Whitewater (rivers, streams)	18	7.9
Don't know	13	5.9
Mosquitoes feed on		
Human blood	207	91.4
Animal blood	133	58.8
Nectar plants/sweet juices from ripe fruits	62	27.4
Water	47	20.8

*Attitudes toward mosquitoes and mosquito control.* Table 4 shows the surveyed gardeners’ attitudes toward mosquitoes and means of control within community gardens. The majority of the respondents thought that the presence of mosquitoes had increased in the past 2 years (56.3%), and only 14.3% had never felt disturbed in the community garden because of the mosquito nuisance. A very large majority of the respondents (81.3%) stated that they were highly or moderately concerned with the presence of mosquitoes in the community garden. Among this group of community gardeners, various reasons for concern were mentioned: bites (62.8%), disease transmission (27.7%), and their beliefs that the majority of mosquitoes in the gardens were Asian tiger mosquitoes (13.8%) and that the growing presence of this species was due to climate change and could cause negative impacts on biodiversity (7.4%). Only 10.5% of the surveyed gardeners believed that the control methods used in their community garden were effective. A five-point Likert scale was used to examine to what extent the gardeners who participated in the survey wanted more information about alternative control methods that could be used against mosquitoes. The average score of 4.16 showed that community gardeners were eager for more knowledge on this issue. Among different sources of information, a majority of participants in the survey answered that they received information about the Asian tiger mosquito through different media, such as newspapers, radio, television, or websites (85.1%). More than half of the participants obtained information through awareness-raising campaigns organized by the Lyon metropolis or by the municipalities in the metropolitan territory (53.1%).

**Table 4:** Mosquito and biological control attitudes in the community gardens

Category	No. of respondents	Proportion within each category (%)
How has the abundance of mosquitoes changed in the past 2 years?		
Increased	134	56.3
Did not change	39	16.4
Decreased	15	6.3
No opinion	50	21
During your activities in the community garden, how frequently have mosquitoes disturbed you?		
Every time	55	23.2
Often	105	44.3
Rarely	43	18.1
Never	34	14.3
Are you concerned about the presence of mosquitoes?		
A lot	110	46.8
A little	81	34.5
Not at all	44	18.7
How do you evaluate the means of control currently used against mosquitoes in the frequented garden?		
Effective	23	10.2
Partially effective	85	37.6
Ineffective	35	15.5
There is no means of control in my community garden	48	21.2
No opinion	35	15.5

*Practices in the community gardens.* Regarding practices, a large majority of the surveyed gardeners (73.2%) conducted their own information research about the Asian tiger mosquito, and in general, this research was carried out online with the use of the internet. Table 5 represents the surveyed gardeners’ practices in community gardens located within the Lyon metropolis territory. Regarding the equipment of their garden plots, a majority of respondents had a hut (88.7%), rainwater collection cans (86%), and a composter (73.3%). Concerning their agricultural practices, it appears that most of the responding gardeners used organic farming (65.1%) and permaculture (45%). A quarter of the respondents reported that they practiced conventional farming. However, only a very small portion of respondents said that they used chemical fertilizers and insecticides (3.7 and 2.1%, respectively). Almost every respondent used organic fertilizers in the community garden (> 90%). The presence of mosquitoes in the community gardens was responsible for a change in habits and practices for a majority of the gardeners in the survey (69.7%). A large majority declared that they protected themselves by using repellents and/or by wearing covering clothing (75.7%). Moreover, 82.1% of the respondents actively fought against the presence of mosquitoes by mechanical methods (e.g., elimination of standing water containers) or by using insecticides. Thus, less than a third (30.3%) of the respondents had not changed any of their habits or practices in the community garden due to the presence of mosquitoes. In addition, among the surveyed gardeners, a quarter (24.8%) stated that they had never tried to reduce the mosquito nuisance or actively used any control methods to prevent the proliferation of the mosquito.

**Table 5:** Practices and effects of mosquitoes on activities in community gardens

Category	No. of respondents	Proportion within each category (%)
Among the following items, your plot contains (multiple answers possible)		
Hut	133	88.7
Rainwater recuperator	129	86
Composter	110	73.3
Flower pot	57	38
No garden equipment	20	13.3
Greenhouse	17	11.3
Planter	41	7.3
Which kind of agricultural practice do you use? (multiple answers allowed)		
Organic farming	157	65.1
Permaculture	109	45.2
Conventional farming	61	25.3
Farming without soil	21	8.7
Don’t know	12	5
In the garden you frequent, which do you use? (multiple answers allowed)		
Organic fertilizers (manure, compost, etc.)	223	92.5
Chemical fertilizers	9	3.7
Insecticides	5	2.1
Did you modify any of your practices in the community garden because of the presence of mosquitoes?		
None	61	30.3
Some	119	59.2
All	21	10.4
Did you perform or limit certain practices to avoid the proliferation of mosquitoes?		
Never	57	24.8
Occasionally	136	59.1
Very often	37	16.1

### How do knowledge, attitudes, and practices influence each other?

In this section, bivariate statistical analysis was used to identify the relationship between gardeners’ knowledge, attitudes, and practices in community gardens. First, we tested the impact of sociodemographic characteristics such as gender, age, or profession on knowledge level, attitude, and practices. No significant relation appeared between gardeners’ profiles and KAP. However, it is interesting that 117 of the community gardeners (87.3%) who completed the survey were highly motivated and willing to participate in the current scientific project on mosquito control by using biological methods. We did not find evidence that any sociodemographic characteristics distinguished these volunteers from the rest of the respondents who had no wish to participate in a mosquito control project. Neither specific knowledge or attitudes nor particular practices in the community garden seemed to explain their willingness to strengthen their contribution to mosquito control. Regarding the statistical relationships between knowledge and practices in the community garden, most of the tested variables showed no significant associations between respondents’ knowledge and their practices. The only significant correlation that we found (Table 6) was that gardeners who had already heard about biological control methods against mosquitoes more often had modified various practices in the garden due to the presence of mosquitoes (80%) than gardeners who had never heard about mosquito control methods (53.9%). Interestingly, the level of concern was significantly correlated with the practices of the survey participants in the community garden (Table 7). For instance, gardeners who were very concerned about mosquitoes were overrepresented in the group of gardeners who modified all or some practices because of mosquitoes (76 and 62% of the respondents in these groups, respectively). In contrast, only 27.9% of the respondents who had not changed their habits in the garden due to the presence of mosquitoes were concerned about the presence of mosquitoes. These results suggest that the more concerned community gardeners are about the presence of the mosquito in their gardens, the more likely they are to take action against this species. In addition, gardeners who were concerned about the mosquito’s presence were overrepresented in the category of respondents who “have often taken action against mosquito proliferation” (60.8% of the 97 respondents in this group). In contrast, the chi-square test showed that gardeners who “have never taken action against mosquito proliferation” were underrepresented (22.8%). Interestingly, the reasons why the respondents were concerned with the presence of mosquitoes (e.g., nuisance, disease transmission or ecological impact) had no significant influence on their practices.

**Table 6:** Relationship between mosquito biological control methods and modification of gardeners’ practices based on the abundance of mosquitoes

Have you changed any of your practices because of the presence of mosquitoes?			
	None, *n* (%)	Some, *n* (%)	All, *n* (%)
Have you ever heard about biological control?			
Yes	4^a^ (10)	32^a^ (80)	4 (10)
No	54^a^ (35.1)	83^a^ (53.9)	17 (11)

Chi-square test

Letter indicates statistically significant differences (^a^*P* < 0.001)

**Table 7:** Relationships between gardeners’ concerns due to mosquitoes and their practices in community garden

Are you concerned about the presence of mosquitoes?			
	Not at all, *n* (%)	A little, *n* (%)	A lot, *n* (%)
Did you modify any of your practices because of the presence of mosquitoes?			
None	22^a^ (36.1)	22 (36.1)	17^a^ (27.9)
Few	4^a^ (0.3)	40 (33.6)	75^a^ (63)
All	1 (4.8)	4 (19)	16^a^ (76.2)
Have you performed or limited certain practices to avoid the proliferation of mosquitoes?			
Never	27^a^ (47.3)	17 (29.8)	13^a^ (22.8)
Occasionally	12 (15.8)	28 (36.8)	36 (47.4)
Often	4^a^ (4.1)	34 (35.1)	59^a^ (60.8)

Chi-square test

Letter indicates statistically significant differences (^a^*P* < 0.001)

Table 8 illustrates the impact of the respondents’ perceptions of mosquito proliferation in the last 2 years on their practices and actions taken against mosquitoes. There was a significant correlation between the perceived increase in mosquito presence and changes in practices in the community gardens, the means of control used in the community garden, and the type of actions taken. For example, the respondents who believed that there was an increase in mosquito presence used more repellents than other gardeners did (20%). In contrast, the level of concern of the respondents regarding mosquito presence had no significant influence on the frequency of their actions against mosquitoes or on the mosquito control methods they used.

**Table 8:** Relationship between the perception of the abundance of mosquitoes over 2 years and its impact on gardeners’ practices.

How has the abundance of mosquitoes changed over the past 2 years?				
	Increased *n* (%)	Did not change *n* (%)	Decreased *n* (%)	No opinion *n* (%)
Did you modify any of your practices because of the presence of mosquitoes?				
None	29^b^ (22)	10 (35.7)	3 (20)	19^b^ (73.1)
Few	85^b^ (64.4)	18 (64.3)	9 (60)	7^b^ (26.9)
All	18^b^ (13.6)	0 (0)	3 (20)	0 (0)
How do you evaluate the means of control currently used against mosquitoes in the frequented garden?				
Effective	5^b^ (4.4)	6 (19.4)	6^b^ (42.9)	6 (18.8)
Partially effective	56 (49.1)	14 (45.2)	6 (42.9)	9^b^ (28.1)
Ineffective	28^b^ (24.6)	3 (9.7)	0 (0)	4 (12.5)
There is no means of control	25 (21.9)	8 (25.8)	2 (14.3)	13^c^ (40.6)
Did you take action against the proliferation of mosquitoes?				
Collectively	49^a^ (49.5)	19^a^ (86.4)	5 (50)	
Individually	50^a^ (50.5)	3^a^ (13.6)	5 (50)	10 (37)
Have you performed or limited certain practices to avoid the proliferation of mosquitoes?				
Never	19^a^ (14.7)	15^a^ (39.5)	20^a^ (40.7)	
Occasionally	43 (33.3)	12 (31.6)	4 (26.7)	17 (35.4)
Often	67^a^ (51.9)	11 (28.9)	8 (53.3)	11^a^ (22.9)
What methods have you already used against mosquitoes? (multiple answers allowed)				
Repellents	48^a^ (20)	5 (12.5)	2^a^ (5)	
Chemical insecticides	1 (0.4)	0 (0)	0 (0)	0 (0)
Elimination of standing water tanks	91 (37.9)	21 (52.5)	9 (50)	18 (45)
Biological control	8 (3.3)	1 (2.5)	1 (5.6)	1 (2.5)
Covering clothing	88 (36.7)	12 (30)	6 (33.3)	13 (32.5)
Other	4^a^ (1.7)	1 (2.5)	0 (0)	6^a^ (15)

Chi-square test

Different letters indicate statistically significant differences (^a^*P* < 0.05; ^b^*P* < 0.001)

The perception of existing mosquito control methods in community gardens could lead them to modify their practices (Table 9). Respondents who believed that mosquito control methods used in the garden were “partially effective” were overrepresented in the category of respondents who changed none or only a few of their activities in the community garden due to mosquitos (42% of the 166 respondents in this group). A significant correlation of this same group was also observed in regard to taking action against mosquitoes. Indeed, 93% of the respondents who believed that mosquito control methods used in the garden were “partially effective” stated that they had taken action against mosquitoes or limited certain practices to avoid the proliferation of mosquitoes. In addition, surveyed gardeners who used no means of control in their gardens were underrepresented in collective action and individual action against mosquito proliferation.

**Table 9:** Influence of the perception of mosquito control methods used in the community garden on gardeners’ practices

How do you evaluate the methods of control currently used against mosquitoes in the frequented garden?					
	Effective *n* (%)	Partially effective *n* (%)	Ineffective *n* (%)	There is no means of control in the garden *n* (%)	No opinion *n* (%)
Did you modify any of your practices because of the presence of mosquitoes?					
None	4 (7)	16^a^ (28.1)	2 (12.3)	18^a^ (31.6)	12^a^ (21.1)
Some	8 (7)	54^a^ (47.4)	22 (19.3)	20 (17.5)	10 (8.8)
All	4 (19)	9 (42.9)	5 (23.8)	2 (9.5)	1 (4.8)
Have you performed or limited certain practices to avoid the proliferation of mosquitoes?					
Never	6 (10.9)	6^b^ (10.9)	4^b^ (7.3)	22^b^ (40)	17^b^ (30.9)
Occasionally	4 (5.3)	33 (44)	16 (21.3)	15 (20)	7 (9.3)
Often	13 (13.8)	46^b^ (48.9)	15 (16)	11^b^ (11.7)	9 (9.6)
Did you take action against the proliferation of mosquitoes?					
Collectively	12 (12.9)	50^b^ (53.8)	16 (17.2)	6^b^ (6.5)	9 (9.7)
Individually	5 (7.5)	24^b^ (35.8)	14 (20.9)	17^b^ (25.4)	7 (10.4)

Chi-square test

Different letters indicate statistically significant differences (^a^*P* < 0.05; ^b^*P* < 0.001)

## Discussion

To our knowledge, this study is the first to collect information on nonprofessional gardeners in community gardens concerning their knowledge, attitudes, and practices in relation to the Asian tiger mosquito in Europe.

This survey allowed us to identify some practices of gardeners that may promote the development of Asian tiger mosquitos in gardens. For instance, 86% of the surveyed gardeners had a rainwater collector on their plot. This kind of watering method is commonly used in community gardens and presents a sustainable and alternative water resource [32, 33]. At the same time, these containers are well adapted for mosquito breeding at the larval stage and are often difficult to protect from mosquitoes (Mariappan, Srinivasan, and Jambulingam [34]). Gardeners should be aware of the ability of this material to serve as a potential breeding site in their garden to take action against mosquito proliferation. Regarding the type of agricultural practices, a quarter of the respondents reported that they performed conventional farming. However, only a very small number used chemical fertilizers and insecticides (3.7% and 2.1%, respectively). Three hypotheses might help to explain this contradiction. First, answers may be biased because many community gardens prohibit the utilization of chemical fertilizers in accordance with the national charter, *Jardin dans tous ses états*, which recommends sustainable cultivation techniques. Second, the surveyed gardeners knew that using chemical fertilizers could have a negative impact on the environment. Thus, even if they used chemical fertilizers, they may have provided what they believed was the expected answer. Third, the surveyed gardeners continued to use conventional agriculture techniques, but they tried as much as possible to avoid chemical inputs and insecticides. According to gardeners’ willingness to be better informed about alternative methods to chemical insecticides, the last hypothesis seems more plausible. This is supported by the fact that most gardeners who would like to be better informed about alternative methods of mosquito control answered that they would be motivated to become involved in specific campaigns for mosquitoes in their garden. This study concludes that all the gardeners who participated in the survey were aware of the presence of the Asian tiger mosquito in the Lyon metropolis. This is consistent with the fact that this mosquito species began to colonize this territory in 2012. Among the main pests in community gardens, mosquitoes were cited most frequently by the surveyed gardeners (72.3%). Regarding knowledge of mosquito characteristics, less than one third of respondents knew that the main physical characteristic of the Asian tiger mosquito is the white dorsal line. Encouraging awareness of specific recognition of the Asian tiger mosquito is essential because of the role of this species in outbreak epidemic events compared to other autochthonous mosquito species.

Most of the respondents were concerned about the presence of the Asian tiger mosquito (90%), mainly because of the mosquito’s annoying bites during diurnal activity (63.8%) rather than its capacity to transmit infections such as arboviruses (27.7%). This finding shows that the gardeners surveyed were concerned with the *Ae. albopictus* as a pest, because of mosquito bites, rather than its ability to transmit disease. This suggests that people tend to be more motivated by the immediate impacts of biting mosquitoes on their quality of life than they are by the threat of diseases. It is human nature for people to respond more to an immediate and tangible problem than to one they perceive as less than immediate or even as hypothetical. This perception seems to emerge from the fact that autochthonous arboviruses remain sporadic, as well as trust in the French health care system [35]. Currently, diseases transmitted by *Ae. albopictus* in Europe are due to sporadic events and mainly depend on imported cases from endemic countries. However, the expected trend in the coming decades is a larger spread of arboviruses [36, 37]. The spread of mosquitoes and mosquito-borne diseases is driven by many factors, including anthropic activities such as global transportation systems, mosquito adaptation in urban areas, and climate change [38, 39]. Recent studies have examined whether people living in arbovirus-endemic countries have better awareness of the risk of arbovirus emergence than people living in nonendemic countries. In previous studies, the European population was compared with the immigrant population from countries where *Aedes*–transmitted diseases are endemic, and the immigrant population had better knowledge and management of this vector species [40, 41]. The population’s awareness is heterogeneous between endemic and nonendemic countries as well as within the country itself due to different ethnic groups and the presence or absence of mosquitoes [41–43]. Changing this perception seems to be an important lever for individual practices that might converge toward mosquito control. Health risk awareness depends on disease susceptibility (which includes the population’s exposure to mosquito-borne diseases) and human perception of the severity of those diseases. With regard to disease susceptibility, the perception of being exposed to mosquito-borne diseases is an important factor to enhance protective measures against mosquitos. In addition, the severity of the disease influences protective practices [42, 43]. In the current survey, respondents were concerned about disturbance by the mosquitos rather than about their capacity to transmit infections. However, this is not linked to a lack of knowledge as 88.5% of the surveyed gardeners knew that mosquitoes can cause disease.

A very large proportion of the respondents (89.5%) declared that they modified their practices because of the presence of mosquitoes (such as time spent on outdoor activities or cancellation of garden events). This is consistent with previous observations showing that *Ae. albopictus* deteriorates people’s quality of life during outdoor activities [44, 45]. These immediate consequences of the biting mosquito deteriorate their activities and motivated gardeners to fight against this pest. This impact on quality of life in community gardens could be a barrier for future urban residents to join a community garden. These findings suggest that gardeners’ community and, to a larger extent, French urban citizens are not prepared to face a potential arbovirus outbreak. Despite the expansion of arboviral diseases in France and on a larger scale in Europe, the European population does not perceive the risk of transmission of these viruses by mosquitoes as a serious problem for the future [35, 40, 46]. Educating people on the dual threat mosquitoes pose in terms biting and disease transmission could have broad implications due to their immediate impact through deterioration of quality of life by bites and the future consequences by proliferation of mosquito-borne diseases.

As expected, better knowledge and protective practices are observed in the general population in endemic areas [40, 47–49]. In this context, knowledge is positively correlated with practices against mosquitoes. In contrast, this study shows that community gardeners’ knowledge has a limited impact on their practices. In fact, the only significant relationship between knowledge and practice is that knowledge of biological control methods used against mosquitos influenced the respondents’ practices. Thus, better awareness of biological control could improve the means used to manage mosquito proliferation. In contrast, other types of knowledge of mosquitoes (e.g., physical characteristics and life-cycle) did not seem to influence the respondents’ practices. This result could be explained by the fact that the study area (mainland France) is not an endemic area. Another plausible explanation is the distribution method of the questionnaires during the survey. Generally, internet surveys lead to a bias of auto-selection (Gingras et al., [50]). Our internet-based survey very likely influenced the characteristics of the respondents according to their educational level and their knowledge of mosquitoes.

In contrast to knowledge, attitudes influence practices. Similar to another KAP survey, this survey highlights significant correlations between attitudes and practices [48]. For instance, the level of concern about mosquito presence modified people’s outdoor activities in the community gardens. The respondents used strategies to avoid mosquitoes such as changing their habits of visiting hours in the garden. According to other KAP surveys and our survey, perceptions of this vector species strongly shape practices, and this link should be considered for future awareness campaigns [48].

The results of this study have some limitations that need to be considered. First, the survey sampling was based on an online distribution that may not properly reflect the community garden population. In this study, all surveyed respondents were included in 30% of the total number of community gardens in the Lyon metropolis, but the profile of the total population of community gardens is still unknown. We can only compare the surveyed population to the Lyon metropolis population estimated in 2018 as no sociodemographic database on community gardens is available [30]. In this study, retired respondents represented 39.6% of the sample population compared to only 21.1% in the Lyon metropolis population. It is very likely that a large part of the garden community participants are retired urban residents; however, we cannot be certain that this group was not overrepresented in this study. A total of 64.9% of the surveyed respondents had a level of education higher than a higher school diploma, compared to 43.2% in the Lyon metropolis population. This could be explained by an overrepresentation of retired people in community gardens, their greater involvement in the associative life of community gardens, or the survey distribution sampling method by email [51]. Second, the questionnaire was written in French, and we know from our fieldwork that non-francophone persons represent a significant proportion in some community gardens. This group of community gardeners might have been underrated in our survey due to language barriers. Third, we compared our study to other surveys in arbovirus-endemic countries, which also differ by socioeconomic context [47, 48, 52, 53]. Such differences can influence the knowledge, attitudes, and practices of the population. Fourth, it is plausible that the surveyed gardeners were more worried about mosquito pests than nonrespondents would be. This is a potential bias linked to the fact that the respondents received information about the study within which the survey took place. Respondents who were concerned about mosquitos in their community gardens were thus likely to be overrepresented in our study.

This KAP survey focusing on the community gardens of the Lyon metropolis allowed us to identify inappropriate practices that can be used as a basis to correct misinformation and/or create an adapted and effective awareness plan. Improving knowledge of the tiger mosquito could be an objective of education plans. As suggested by our findings, mosquitoes are perceived as nuisance pests rather than disease vectors. Changing the perception of gardeners could induce better control of their behavior against mosquitoes. In a general context of increasing green areas in cities and a greater risk of outbreaks of *Aedes*-transmitted diseases, this survey shows a significant lack of knowledge and awareness of practices to help control the Asian tiger mosquito proliferation among people who take a particular interest in nature. Pest proliferation in urban green spaces needs to be better understood by society and political stakeholders [54]. This study is the cornerstone to enhance pest integrative management in green urban areas. In addition, community gardens are spaces of racial/ethnic diversity and community sharing [55]. The richness of different cultures in community gardens could contribute to improving awareness and could be the key to an efficient educational plan in gardens related to community engagement (e.g., sharing knowledge, preventive practice). Further studies are needed to implement similar surveys in other cities where *Ae. albopictus* has been present for a longer period to compare perceptions of the Asian tiger mosquito and to determine any awareness-raising operations that have been established.

## Supplementary Information

**Additional file1: **Survey of the collective gardens of the metropolis of Lyon.
