# Supplementary material for: How do attitudes shape protective practices against the Asian tiger mosquito in community gardens in a nonendemic country?
Source: Parasit Vectors. 2022 Nov 22;15:439. doi: 10.1186/s13071-022-05520-3 (PMC9682734; doi:10.1186/s13071-022-05520-3)
Supplement: Supplementary file 1 — Additional file1: Survey of the collective gardens of the metropolis of Lyon. [file 13071_2022_5520_MOESM1_ESM.pdf]

Survey of the collective gardens of the metropolis of Lyon

The presence of many green spaces in urban areas is a source of biodiversity and well-being for the inhabitants, but can also contribute to the proliferation of certain harmful species.

As part of my first year of thesis in biology at the University of Lyon 1, I am carrying out a survey on practices in collective gardens in urban areas and the nuisances due to certain species that colonize these gardens.. The metropolis of Lyon, with more than 200 collective gardens to date, is my field of study..

Ultimately, the goal of this study is to find new means of pest control that are more sustainable, better targeted and respectful of the environment..

Beforehand, I set up a survey of the members of the collective gardens of the Metropolis, in order to know the frequentation of these gardens, the cultivation practices as well as any practices put in place to fight against pests.

The questionnaire lasts 15 min, thank you in advance for your participation.

Survey designed by Pénélope Duval, PhD student at the UMR d'Ecologie Microbienne and the UMR Environnement Ville et Société, funded by the Ecole Urbaine de Lyon. In accordance with the regulations in force regarding the protection of personal data, your answers will remain strictly confidential and anonymous..

I undertake to use them only in the context of my research and not to distribute them to any person, structure or outside company.. The information collected in this form is recorded in a computerized and secure file on the servers of the TGIR Huma-num and at the LEM (UMR 5557). They are intended for my doctoral work. You have a right of access, rectification, portability of information concerning you, and limitation of the processing of your data.

To assert your rights or report errors: penelope.duval@universite-lyon.fr

Attendance and practices within a collective garden

When did you join or participate in activities in a collective garden for the first time?

- ◯ Less than 1 year
- ◯ Between 1 and 5 years
- ◯ Between 6 years and 10 years
- ◯ More than 10 years

**If you participate in several collective gardens at the same time, you will answer taking into account the garden you frequent the most.*

Since when did you join the collective garden in which you are currently (indicate the number of months or years)?

What is the name of the garden you are currently frequent?

In which municipality or district is this garden located?

Who is the manager of this collective garden?

- ◯ A social landlord (for example GrandLyon Habitat)
- ◯ The town hall
- ◯ An association
- ◯ The garden is self-managed
- ◯ Don't know
- ◯ Other

Other, give details

What is your role in this collective garden? (many possible responses)

- ☐ Initiator of the project
- ☐ Participant/Gardener
- ☐ Developer
- ☐ Financial partner
- ☐ Animator
- ☐ Professional market gardener
- ☐ Does not wish to answer
- ☐ Other

Other: specify

In this garden, what activities do you practice? (many possible responses)

- ☐ Planting of fruits and/or vegetables
- ☐ Planting of ornamental plants and/or flowers
- ☐ Participation in workshops
- ☐ Participation in pedagogical workshops
- ☐ Manufacture of garden furniture
- ☐ Other

Other: specify

What were your motivations for joining a collective garden? (many possible responses)

- ☐ To grow fruits and vegetables yourself
- ☐ For the pleasure of gardening
- ☐ To practice physical activities
- ☐ To create social connections
- ☐ To practice outdoor activities
- ☐ To transmit gardening practices to the youngest
- ☐ Other

Other: specify

In which season(s) do you visit the collective garden the most? (many possible responses)

- ☐ Autumn
- ☐ Winter
- ☐ Spring
- ☐ Summer
- ☐ No difference in attendance depending on the season

At what time(s) of the day do you go to the collective garden the most? (many possible responses)

- ☐ Morning
- ☐ Lunchtime
- ☐ Afternoon
- ☐ Evening
- ☐ No difference in attendance

During the season(s) when you visit the collective garden, you go there:

- ☐ Every day
- ☐ Several times a week
- ☐ Once a week
- ☐ Once every two weeks
- ☐ Once a month
- ☐ Other

Other, give details

How long do you stay on average in the collective garden ?

- ○ Less than 30 min
- ○ Between 30 min and 1h
- ○ Between 1h and 2h
- ○ More than 2h

The type of plot you cultivate is:

	Yes	No	Don't know
An individual plot	○	○	○
A collective plot	○	○	○

If you have one or more individual plots, how many do you have?

- ○ 1
- ○ 2
- ○ 3
- ○ More than 3

Do you know the area of your individual plot(s)?

- ◯ Yes
- ◯ No

If so, what is its area (in square meters)?

[ ]

Is your plot fully cultivated?

- ◯ Yes
- ◯ Don't know
- ◯ No

If not, how is the rest of your plot laid out?

[ ]

Among the following items, your plot contains: (multiple answers possible)

- ☐ No garden furniture
- ☐ Planter
- ☐ Hut
- ☐ Composter
- ☐ Greenhouse
- ☐ Rainwater recuperator
- ☐ Flower pot
- ☐ Other

Other: specify [ ]

Do you know the area of the garden you frequent?

- ○ Yes
- ○ No

If so, what is its area (in square meters)?

[ ]

Among the collective facilities, the garden you frequent contains: (several answers possible)

- ☐ No collective furniture
- ☐ Dry toilets
- ☐ Collective hut
- ☐ Greenhouse
- ☐ Composter
- ☐ Informative panel
- ☐ Barbecue
- ☐ Rainwater collector
- ☐ Other

Other: specify [ ]

Which of the following do you use as an agricultural practice: (several answers possible)

- ☐ Conventional farming
- ☐ Organic farming
- ☐ Farming without soil
- ☐ Permaculture
- ☐ Don't know
- ☐ Other

Other: specify [ ]

In the garden you frequent, do you use: (multiple answers possible)

- ☐ Chemical fertilizers (nitrogen fertilizers, phosphate fertilizers, etc.)
- ☐ Organic fertilizers (manure, compost, etc.)
- ☐ Insecticides
- ☐ Other

Other: specify [ ]

If you use fertilizers or insecticides, do you know the name?

○ Yes ○ No

If yes, specify the name

[ ]

Do you think that the air pollution of the city influences the pollution within your garden?

○ Not at all ○ A lot of
○ A little ○ No opinion

Have you ever had soil analyzes done on your plot or in the collective garden?

○ Yes ○ No

If yes, what type of analysis did you perform?

[ ]

If you have carried out soil analyses, would you be willing to send us the results in order to facilitate our field work?

○ Yes ○ Do not wish to answer
○ No

To water your plants, do you use: (multiple answers possible)

☐ I don't water ☐ Rainwater (water collector)
☐ City water ☐ Other
☐ Drilling

Other: specify [ ]

How do you water your plants? (several answers possible)

- [ ] Watering can
- [ ] Drip irrigation
- [ ] Water jet
- [ ] Other

Other: specify ____________________

Nuisances

In gardens, many plant or animal species can have adverse effects on your crops or on your well-being. In this part, we will consider only animal pest species.

In the collective garden that you frequent, have you encountered one or more harmful species (causing harmful effects on your crops or on your well-being) among the following: (several answers possible)

- ☐ Aphids
- ☐ Snails
- ☐ Slugs
- ☐ Birds
- ☐ Colorado potato beetles (doryphore)
- ☐ Mosquitoes
- ☐ Rodents
- ☐ Ticks
- ☐ Other

Other: specify

Do you find that the presence of mosquitoes in the past two years:

- ○ Increased
- ○ Did not change
- ○ Decreased
- ○ No opinion

During what period(s) of the year do you think mosquitoes are present? (many possible responses)

- ☐ January-April
- ☐ May-June
- ☐ July-August
- ☐ September-October
- ☐ November-December
- ☐ No opinion

During your activities in your collective garden, mosquitoes have bothered you:

- ○ Every time
- ○ Often
- ○ About half the time spent in gardens
- ○ Rarely
- ○ Never

If mosquitoes bothered you, was it: (several answers possible)

- ☐ To the mosquitoes that hover around me
- ☐ To the bite itself
- ☐ To the very large number of bites
- ☐ To my reaction to the bites (swelling, itching, etc.)
- ☐ Other

Other: specify

Are you concerned about the presence of mosquitoes?

- ○ Not at all
- ○ A little
- ○ A lot

If so, why are you concerned about the presence of mosquitoes?

Have you changed any of your practices in the collective garden because of the presence of mosquitoes?

- ○ None
- ○ Some
- ○ All

If you answered 'none' to the previous question, you can go directly to the question: 'Within the collective garden, have you acted or limited certain practices to prevent the proliferation of mosquitoes?'

How have you changed your practices within the collective garden?

Have you changed the period when you go to the collective garden ?

- ○ Yes
- ○ No
- ○ No opinion

Is this a change in your hours?

- ○ Yes
- ○ No

If yes, specify

Is it a change in the period/season?

- ○ Yes
- ○ No

If yes, specify

Is this a change in something else? (specify)

Within the collective garden, have you acted or limited certain practices to avoid the proliferation of mosquitoes?

- ○ Never
- ○ Rarely
- ○ Occasionally
- ○ Quite often
- ○ Very often

If you have acted against mosquitoes, can you explain how you fight against them?

If you have acted against mosquitoes, how often do you act to prevent the proliferation of mosquitoes in the garden?

If you have acted against mosquitoes, is it individually or collectively?

- ○ Individually
- ○ In a group
- ○ With another person
- ○ Other

other, give details

If you took action against mosquitoes, how and with whom did you take action?

To fight against mosquitoes, you have already used during the past year in the collective garden(s): (several answers possible)

- ☐ Repellents
- ☐ Covering clothing
- ☐ Chemical insecticides
- ☐ Biological control (eg predators, hormones, etc)
- ☐ Elimination of standing water tanks
- ☐ Other

Other(s), specify

The Asian tiger mosquito

Have you ever heard of the tiger mosquito?

- ◯ Yes
- ◯ No
- ◯ Don't know

If yes, by what means were you informed? (several answers possible)

- ☐ Through the media (newspaper, radio, television, news site, etc.)
- ☐ Through a communication campaign (Lyon metropolis, the city of Lyon, your municipality, etc.)
- ☐ Through the organization managing your collective garden(s) (for example an association)
- ☐ Through awareness campaigns (ARS, EID, etc.)
- ☐ Other

Other: specify

Have you ever researched information about the tiger mosquito on your own?

- ◯ Yes, often
- ◯ Yes, it has happened before
- ◯ No, never

If yes, you searched by: (multiple answers possible)

- ☐ Internet
- ☐ Book
- ☐ Via the services of your municipality
- ☐ Via the services of the Lyon metropolis
- ☐ Other

Other: specify

In your opinion, the tiger mosquito is: (multiple answers possible)

- ☐ A pest (via bites)
- ☐ A useful species in the food chain
- ☐ A species of mosquito that can transmit infectious agents responsible for diseases in humans and animals
- ☐ An invasive species
- ☐ A species of mosquito like the other
- ☐ Other
- ☐ Very present on French territory (mainland)

Other: specify

Do you think you recognize the tiger mosquito among other mosquito species?

- ○ Yes
- ○ Don't know
- ○ No

If yes, according to which criterion(ies): (several answers possible)

- ☐ The size
- ☐ The hours of observation
- ☐ The stripes on the legs
- ☐ The wings
- ☐ The dorsal
- ☐ Other

Other: specify

According to you, the tiger mosquito develops in: (several answers possible)

- ☐ Large body of water (lakes, marshes, etc.)
- ☐ Whitewater (rivers, streams, etc)
- ☐ Vegetation
- ☐ Don't know
- ☐ Medium and small stagnant water reservoir (watering can, cup, rainwater collector, etc.)
- ☐ Other
- ☐ Air

Other: specify

Do you think mosquitoes feed on: (multiple answers possible)

- ☐ Water
- ☐ Sweet juice from ripe fruits
- ☐ Human blood
- ☐ Other insects
- ☐ Blood collected from other animals
- ☐ Other
- ☐ Plant

Other: specify ______

Would you like to be better informed about this species of mosquito?

- ○ Yes
- ○ Don't know
- ○ No

If yes, how would you like to be informed? (many possible responses)

- ☐ Local press
- ☐ By internet (email, social networks)
- ☐ National press
- ☐ The metropolis or the commune
- ☐ Radio
- ☐ Manager of the collective garden
- ☐ Directly in the gardens
- ☐ Other

Other: specify ______

Biological control

How do you evaluate the means of control currently used against mosquitoes in the frequented garden?

- ○ Effective
- ○ Partially effective
- ○ Ineffective
- ○ To my knowledge, there is no means of control in the garden that I frequent
- ○ No opinion
- ○ Other

other, give details [ ]

Among the methods of mosquito control, have you ever heard of biological control?

- ○ Yes
- ○ No
- ○ Don't know

If yes, can you, in a few words, explain what it consists of?

[ ]

On a scale of 1 (I don't want to be informed) to 5 (I want to be better informed) would you like to be better informed about alternative methods to the use of chemical insecticides?

[ ]

Within your collective garden, would you be ready to become an actor in the fight against the spread of the tiger mosquito?

- ○ Yes
- ○ No
- ○ No opinion

If so, what degree would you like to invest in?

- ○ A little
- ○ A lot
- ○ Very much

If you wish, you can leave your contact details below in order to receive the results of the survey and/or participate in the follow-up to my field study.

Descriptive data

In which city/district do you live?

Sex

- ◯ Male
- ◯ Female
- ◯ Other

Age

- ◯ < 18 years old
- ◯ 18-25 years old
- ◯ 25-35 years old
- ◯ 35-50 years old
- ◯ 50-65 years old
- ◯ 65-75 years old
- ◯ > 75 years old

Status

- ◯ Single
- ◯ Couple without children
- ◯ Couple with child(ren)
- ◯ Other

Other, give details

Professional life

- ◯ In activity
- ◯ In training
- ◯ In study
- ◯ Seeking employment
- ◯ No activity
- ◯ Retired
- ◯ Does not wish to answer
- ◯ Other

Other, give details

You are :

- ◯ Employed full-time
- ◯ Employed part-time
- ◯ Other

Other, give details

What is or was your profession (before you retired)?

- ◯ Farmer
- ◯ Employee
- ◯ Worker
- ◯ Executive
- ◯ Self-employed worker
- ◯ Other

Other, give details

What is your education level ?

- ◯ Level below high school diploma
- ◯ Level of high school diploma
- ◯ Level above high school diploma
- ◯ Other

Other, give details

Finally, do you have any general comments or suggestions (including on the questionnaire)?

Thank you for the time you spent on this survey!

UNIVERSITÉ DE LYON | Lyon 1 Le PASSE JARDINS ECOLOGIE MICROBIENNE LYON UNIVERSITÉ LUMIÈRE LYON 2
